# Parental perceptions of the psychosocial outcomes of equine-assisted activities and therapies for children with autism spectrum disorder in Japan: a phenomenological study

**DOI:** 10.1080/17482631.2025.2585638

**Published:** 2025-11-19

**Authors:** Namiko Kawamura, Mayu Sakamoto, Chikako Hashimoto, Yuji Ozeki, Kayoko Machida

**Affiliations:** a School of Nursing, Shiga University of Medical Science, Otsu, Shiga, Japan; b School of Medicine, Shiga University of Medical Science, Otsu, Shiga, Japan; c Sapporo City University, Sapporo, Hokkaido, Japan

**Keywords:** Equine-assisted activities and therapies, autism spectrum disorder, psychosocial development, parental perspective, qualitative research, phenomenology, well-being, nursing

## Abstract

**Background:**

Children with autism spectrum disorder (ASD) often experience challenges in social communication, behavioral regulation, and daily life adaptation. Equine-assisted activities and therapies (EAATs) have been implemented as complementary approaches to support psychosocial development. However, little is known about the experiences of parents in Japan whose children participate in EAATs.

**Aim:**

The study aims to qualitatively explore how parents in Japan perceive the psychosocial outcomes of EAATs for their children with ASD. By focusing on parental perspectives, this study seeks to clarify how EAATs may contribute to the well-being of both children and their families, and to inform the development of supportive therapeutic environments.

**Methods:**

This qualitative study employed a phenomenological approach. Semi-structured interviews were conducted with ten parents whose children with ASD had participated in EAATs for a minimum of six months. Thematic analysis was guided by Colaizzi’s method.

**Results:**

Four primary developmental stages emerged from the analysis of children’s experiences: (1) Learning physical and mental harmony, (2) Exploring interests and strengthening bonds with oneself and others, (3) Inspiring independence and interaction, and (4) Building resilience and communication skills. Furthermore, parents reported three overarching outcomes from their own involvement: (1) Parental well-being and engagement through horseback riding, (2) Parental joy and recognition of their child’s growth, and (3) Fostering Family Connection and parent-to-parent interaction. Participant narratives illustrated these themes vividly, highlighting specific examples of behavioral and emotional change.

**Conclusions:**

EAATs were perceived by parents as fostering not only the psychosocial growth of children with ASD but also enhancing parental well-being and family cohesion. To support children’s psychosocial development and promote the health and well-being of their parents, nurses could play a distinctive role by observing both psychological and physical changes, monitoring how these influence daily functioning and interpersonal relationships, and supporting parents in interpreting their child’s developmental progress.

## Introduction

### Autism spectrum disorder and the need for long-term support

Children with autism spectrum disorder (ASD) experience persistent challenges in communication, social interaction, and adapting to environmental changes. These difficulties often cause stress in everyday life and can lead to secondary problems, such as heightened anxiety, sleep disturbance, and depressive symptoms (American Psychiatric Association, 2022).

In Japan, the number of children requiring support has increased markedly, with 8.8% of elementary and junior high school children in regular classrooms reported to experience significant learning and behavioral difficulties (Ministry of Education, Culture, Sports, Science and Technology, Japan, 2022). Furthermore, the prevalence of developmental disabilities in Japan has increased seven-fold over the past 20 years. As of 2022, over 140,000 children use after-school day services and other support programs (Ministry of Health, Labor and Welfare, Japan, 2022). Kugimiya (2016), a child psychiatrist in Japan, emphasized the need for medical support that not only understands these children's characteristics but also respects their individuality. Comprehensive, continuous, and family-centered interventions are needed.

### Equine-assisted activities and therapies in international research

Equine- assisted activities and therapies (EAATs) have emerged as a comprehensive approach that integrates physical, psychological and social elements. EAATs have been reported to have positive effects on mental health (Umbarger, 2007; Wood et al., 2021). A growing body of evidence, mainly from Europe and North America, has reported psychosocial benefits for children with neurodevelopmental conditions, including ASD and ADHD. Reported benefits for children with ASD include enhanced emotional stability and improvements in both verbal and nonverbal communication. Gabriels et al. (2015) observed increases in the total number of words and new words spoken in ASD children who took part in 10 weeks of EAATs. In a subsequent study, Gabriels et al. (2018) reported significant improvements in social and communication behaviors. Llambias et al. (2016) found that EAAT were associated with increased smiling, laughing, and singing behaviors. Tan and Simmonds (2018) reported improvements in children’s self-concept and self-regulatory abilities. Peters et al. (2021) reported improved in social motivation. Zhao et al. (2021) found that, compared with controls, children showed significant gains in overall social skills and communication.

More recently, qualitative studies have highlighted parental and children’s perspectives. Using thematic analysis, Kalmbach et al. (2020) demonstrated that EAATs benefited both the child and the family as improvements in mood or social skills in children positively influenced parents and other family members. Carlsson et al. (2024) described parents of adolescents with mental illness perceived EAATs as gradually increasing confidence “bit by bit,” illuminating processes of increased, leading to greater harmony, enhanced self-identity, and improved capability. Punzo et al. (2022) also claims that children experienced feelings of relief from everyday stress and anxiety, nurturing self-esteem, and strengthened their sense of self-worth. These findings highlight the importance of examining how EAATs benefit not only children buy also their families.

Although previous studies several studies investigated valuable insights, gaps in knowledge still exist. First, most previous studies investigated physical outcomes such as changes to posture, motor coordination, and muscle tone; on the other hand, psychosocial dimensions have received less systematic attention. Second, methodological diversity such as variations in participant characteristics, intervention duration, and settings, makes it difficult to identify the mechanisms underlying positive change (Kawamura et al., 2024). Third, few studies have examined EAATs in the cultural and healthcare context of Asian countries, leaving an important geographic gap in the literature.

### EAAT research and challenges in Japan

Research on EAATs in Japan has focused mainly on physical outcomes. Ishii et al. (2017) reported that horseback riding helped improve children’s posture, particularly by encouraging a straight back. Miwa et al. (2015) found a correlation between horseback riding and changes in behavior at home among children with pervasive developmental disorders. However, Takisaka (2011) reported challenges in developing effective training methods due to the shortage of qualified trainers, therapists, and therapy-suitable horses in Japan. Kawamura et al. (2015) reported improved communication skills in little children with ASD or ADHD during EAATs in a preliminary observational study. Further studies are needed with regard to the utilization of EAATs in Japan.

Clinical perspectives in Japan emphasize the need for psychosocial support that respects each child’s individuality while addressing secondary issues such as communication and emotional regulation (Kugimiya, 2016). However, little is known about how EAATs contribute to these domains, particularly from the perspective viewpoint of parents who observe their children’s development in daily life. From a nursing perspective, it important to support the physical and psychosocial aspects of children’s health and family well-being. In Japan, nurses often play central roles in daycare centers and community health, particularly in managing physical and mental health symptoms and monitoring quality of life (QOL). Therefore. their involvement in welfare-related EAAT programs could potentially enhance interdisciplinary collaboration and holistic care.

Against this background, it is useful to clarify parents’ perceptions of EAATs. Their insights can provide valuable insights into how these interventions affect children’s functioning and family adaptation and resilience. These perspectives can inform practice, guide training for professionals, and contribute to the development of sustainable therapeutic environments.

## Aim of the study

This study aimed to qualitatively investigate how parents in Japan perceive the psychosocial outcomes of EAATs for their children with ASD. By focusing on parental perspectives, this study seeks to investigate how EAATs contribute to the well-being of children and their families, and to inform the development of supportive therapeutic environments.

## Methods

### Design

A qualitative research design rooted in phenomenology was adopted to explore the lived experiences of parents. Taguchi (2014), influenced by Husserl, describes phenomenology not as an activity that aims obtaining a bird's-eye view, but rather an activity that seeks to engage with phenomenological thinking itself from a particular perspective. It is an approach that aims to uncover the hidden and often overlooked dimensions that rarely enter our thematic awareness because they are so self-evident.

### Participants

We recruited parents of children diagnosed with ASD, ranging from infancy to high school age, who had been regularly participating in medical and welfare services, that included EAATs (including horseback riding) at least twice a month for a continuous period of six months or more. The recruit the patients, the researchers requested the cooperation of the directors of after-school day service centers, who identified and approached parents who were considered able to participate in interviews and met the inclusion criteria. Ten recruited parents agreed to participate. Prior to participation, the researchers provided participants with a full explanation of the study and obtained their written informed consent.

### Data collection

Semi-structured interviews were conducted between February and November 2022. Each participant completed two interviews, lasting 65–108 minutes in total. All interviews were recorded. The interview began with the question: “Can you tell me how EAATs worked for your child?” and “Can you tell me what EAATs mean for you?” Based on the participants’ stories, in-depth questions were used. Further questions were asked about the child's symptoms, emotions, relationships, and environment.

## Data analysis

Data were analyzed based on Colaizzi’s phenomenological method (Colaizzi, 1978) using the following four steps to derive themes:

1. All narratives obtained from participants were read thoroughly in order to grasp their meaning, with the researchers suspending their preconceptions.
2. Significant statements reflecting parents’ perspectives on their child’s horseback riding, EAATs and their own lived experiences of engaging with the child in this context were extracted. Meanings were formulated from these statements, while also attending to implicit or unspoken aspects of the narratives. Creative insights were applied to illuminate meanings embedded in the participants’ contexts and horizons.
3. This procedure was repeated, and clusters of themes were generated from the aggregate of formulated meanings.
4. The findings were integrated into a comprehensive description and presented in a clear, coherent narrative.

All analytical procedures were independently performed by three researchers experienced in phenomenological methods. Discrepancies were examined and consensus was reached through discussion regarding the interpretation of meanings.

## Results

### Participants

Ten parents (aged 37 to 49) participated in the study. Of these, two were fathers and eight were mothers. Each participant was the parent of one child (aged 7–14; one girl, nine boys) who had been diagnosed with ASD in early childhood and was attending elementary or junior high school, at the time of the study. The children attended horseback riding activities every 1–2 weeks for 0.5–7 years.

EAATs were provided as an after-school daycare service that was a part of welfare services for persons with disabilities in Japan. Data were obtained through two 65–108-minute interviews with each participant (Table I). The transcripts of the participants’ oral descriptions were categorized based on similarities by three qualitative researchers.

**Table I.** Demographic information of participating parents and contextual details of their children.

Participant	A	B	C	D	E	F	G	H	I	J
Relationship with the child	Mother	Mother	Mother	Father	Mother	Mother	Mother	Mother	Mother	Father
Child’s age and sex	14, Male	8, Male	10, Male	12, Male	11, Male	13, Male	11, Female	7, Male	9, Male	8, Male
Child’s other diagnoses/functional abilities	－	－	AD/HD, LD	AD/HD	－	－	ID	－	AD/HD, ID	－
Frequency, Length of EAATs participation	1−2/month, 7 years	2/month, 6 months	2/month, 2 years	2/month, 2years	2/month, 5years	1−2/month, 7 years	2/month, 3.5 years	2−4/month, 2-3years	2/month, 3years	1−2/month, 3years
Total interview time (min)	78	65	108	72	85	66	88	88	89	85

AD/HD: Attention-Deficit/Hyperactivity Disorder; LD: Learning Disabilities; ID: Intellectual Disability.

### The effects of long-term EAATs on children as perceived by their parents

As a result of the qualitative analysis of all data of 208 statements, two-dimensional aspects of the long-term impact of EAATs for children with ASD were identified by their parents. A total of 156 statements were extracted regarding children’s experiences with long-term EAATs as perceived by their parents. From these, four overlapping main stages and 18 sub-themes were identified. Furthermore, 52 statements and 10 sub-themes were identified regarding the parents’ own experiences (Table II). Detailed information is provided in Supplementary Table S1.

**Table II.** Two-dimensional aspects of the long-term impact of EAATs for children with ASD as perceived by their parents, and sub-themes.

Dimension	Themes	Sub-themes	Number of statements
Children's horseback riding experiences as perceived by parents	Learning Physical and Mental Harmony	Enjoyment and sense of security in horseback riding	22
		Reassurance from professional staff support	16
		Core stability	10
		Improvement in smoothness of movement	8
		Established as part of a daily routine	7
		Positive effects on work tasks (increased focus)	2
		Enhanced digestive function	1
	Exploring Interests and Strengthening Bonds with Oneself and Others	Expanded interest and empathy toward others	19
		Improved confidence through riding	16
		Increased confidence through interaction with children of different ages	4
		Broadened interest in horses and other animals	3
		Improved planning skills	2
		Opportunities to learn new tasks	1
	Inspiring Independence and Interaction	Expanded actions in daily life	9
		Increased motivation for riding	8
		Improved conversational and self-expression skills	7
	Building Resilience and Communication Skills	Improved ability to cope with changing situations	16
		Expanded communication with diverse people through horseback riding	5
Parental Experiences in Supporting Their Child’s Horseback Riding	Parental Well-Being and Engagement Through Horseback Riding	Parental refreshment (nature and healthy distance from the child)	9
		Reassurance from staff's professional support (specifically for parents)	6
		Peace of mind from observing their child’s well-being	5
		Increased interest in horseback riding through the child’s involvement	2
		Stability in parents' routines through transport arrangements	1
	Parental Joy and Discovery in Their Child’s Growth	Joy in witnessing the child’s growth as a parent	8
		Discovering new strengths in their child as a parent (impressed)	8
	Fostering Family Connections and Parent Interaction	Adjusting family dynamics	6
		Opportunities for parent interaction	4
		Opportunities for exchanging information among parents	3

Based on the dimensional aspects of children’s horseback riding experiences as perceived by parents, the following themes were identified.

- Learning Physical and Mental Harmony
- Exploring Interests and Strengthening Bonds with Oneself and Others
- Inspiring Independence and Interaction
- Building Resilience and Communication Skills

Based on the dimensional aspects of parental experiences in supporting their child’s horseback riding, the following themes were identified.

- Parental Well-being and Engagement through Horseback Riding,
- Parental Joy and Discovery in Their Child’ Growth,
- Fostering Family Connections and Parent-to-Parent Interaction.

### Learning physical and mental harmony

This theme included the following subcategories: Enjoyment and sense of security in horseback riding, Reassurance from professional staff support, Core stability, Established as part of a daily routine, Improvement in smoothness of movement, Positive effects on work tasks (increased focus), and Enhanced digestive function.

Parents felt that horseback riding increased their children’s sense of enjoyment. They also recognized that continued participation led to improvements in core strength, coordination between vision and movement, and overall body awareness. In addition, even for children who found it difficult to express their emotions, parents perceived that they were able to build trusting relationships with the staff—who held medical and welfare qualifications—and develop meaningful connections with the horses. Parents perceived that their children were genuinely engaged in riding and acted with their own intention.

“*He doesn’t want to go to school, so he really dislikes when it’s time to leave. It seems like he just doesn’t want that moment to come, so unless we tell him, 'It’s time to go!' he won’t get ready. But with the pony sessions, it’s the opposite—he’ll be the one saying, ‘Dad, we’re going to be late! Let’s go!*’” (Participant D)

“*When we go to unfamiliar or fun places, my son tends to get really excited—like, waaah! Especially if friends are around, his energy really goes up, and, well, he’s often been told to calm down since he was little. But here, it’s more about interacting with the instructors rather than with friends, so he doesn’t get overly excited like that.*” (Participant H)

### Exploring interests and strengthening bonds with oneself and others

This theme includes the following sub-themes: Expanded interest and empathy toward others, Improved confidence through riding, Increased confidence through interaction with children of different ages, Broadened interest in horses and other animals, Improved planning skills, and Opportunities to learn new tasks.

Parents noted that the children began to demonstrate an understanding of the feelings of horses, a skill they acquired from the staff. This understanding was also reflected in their actions such as helping younger children and showing consideration and empathy toward others. Through these interactions, parents felt that their children were gaining confidence by caring for younger children. Even when things did not go as planned or their moods changed, the children continued to interact with their classmates.

“*With animals, unless you take the initiative, they won’t really respond to you, right? I don’t think you get much back from them, but it still makes you want to reach out and interact with them yourself, and I think that’s a really good thing for my son.*” (Participant I)

“*My son had only experienced being helped by others, not helping others himself. But, after coming here, caring for the animals became his main focus. I began to see new feelings in him, like wanting to take care of others and be kind to animals. It was the first time I had seen this side of him, and I believe it emerged through his direct interaction with the animals in horse therapy.*” (Participant E)

### Inspiring Independence and Interaction

This theme includes the following sub-themes: Expanded actions in daily life, Increased motivation for riding, and Improved conversational and self-expression skills.

Parents shared how their children were able to enjoy humorous interactions with other children and staff, building on the confidence they had gained. They also mentioned how their children had developed the ability to share their own achievements, such as riding horses and succeeding in horse-riding competitions, with other children. This helped to increase their motivation to set higher goals.

“*In my child’s case, I think at first, he probably didn’t like cleaning up the horse’s poop. But now, he does it without complaining. I feel like that might have reduced his resistance to cleaning in general—even at home. So having stable cleaning as part of the schedule seems like a really good thing.*” (Participant D)

“*He’s not just passive anymore—he’s started to take initiative and ask things like, “What should I do here?” or “Is it okay if I do that?” Not only at school, but also with people who aren’t his teachers or parents, he seems to be able to speak up on his own.*” (Participant I)

### Building resilience and communication skills

This theme includes the following sub-themes: Improved ability to cope with changing situations and Expanded communication with diverse people through horseback riding.

The parents spoke about how their child, in horse-riding activities, was able to stay calm and adapt to changes instead of panicking when things did not go as planned. They also said that their child showed perseverance not only in horse riding but also in other school and club activities, which they recognized as being influenced by their involvement in horse riding. They shared that, through long-term participation in horse riding, their child's relationships at school had become more stable.

“*My child never really had intense meltdowns to begin with. When he did, it was only in response to something completely unexpected, like, for example, if he accidentally walked into a spiderweb, then it would be a huge reaction. But now, he’s become much more flexible. Instead of a full-blown panic, it might just end with him being on the verge of tears or him being able to hold it together. For instance, if he was told he would be the first to ride but another child asked, 'Sorry, would you mind switching places with me?' my son would now say, 'Sure,' and let them go ahead.*” (Participant C)

“*She mentioned how it was fun when older boys were kind to her and things like that. So I feel like, for her, it wasn’t just the horses—the chance to connect with different people was also really good for her.*” (Participant G)

### The parents' own experience of supporting their children's long-term horseback riding

From the data, four themes and 11 sub-themes were extracted that reflected parents' experiences and concerns about their children participating in horseback riding.

#### Parental well-being and engagement through horseback riding

This theme includes the following sub-themes: Parental refreshment (nature and healthy distance from the child), Peace of mind from observing their child’s well-being, Reassurance from staff's professional support (specifically for parents), Increased interest in horseback riding through the child’s involvement and Stability in parents' routines through transport arrangements.

Parents talked about how they noticed changes in their children that they would not normally see through the process of picking up and dropping off their children, and how this made them happy as they realized their children were maturing.

“*I like animals, so being here helps me relax, even when I’m tired from work or housework. My son enjoys it too, so it’s a great stress reliever for both of us.*” (Participant J)

“*By coming here, I can get advice from the teachers. And from my point of view, there are parts of my son that feel really tough or difficult to cope with. (…) Having a place where I can talk if something comes up, a place where I can say what I need to say —that’s something that’s been really important for us, ever since the early therapy days when I’d always talk with other moms.*” (Participant C)

### Parental joy and recognition of their child’s growth

This theme includes the following sub-themes: Joy in witnessing the child’s growth as a parent and Discovering new strengths in their child as a parent (impressed).

The parents were able to see their children interacting with the staff and other children through horse riding, observing the children looking after the horses, and seeing their children in a different light to how they were at home. They were also able to see how their children interacted with their friends at school and were able to renew their perceptions of their children. The parents were happy to see their children maturing.

“*He’s still not very good at moving his body. And when I see little kids around our neighborhood, like 3- or 4-year-olds, starting to ride bikes or jump rope, I can’t help but think, “Why can’t he do that?” Even though I don’t say it out loud, I still think it. But then, I start to notice the things he’s working hard on. And because of that, I think, ‘Oh, he’s definitely growing,’ and it reminds me that there’s no way he won’t grow. It makes me think I should take things a little slower, and approach things in a way that suits him better. Every time we come here, I end up thinking this way.*” (Participant H)

“*There are competitions here as well. The staff at this facility make sure to praise and encourage the children, giving out awards to everyone. When this happens, he looks genuinely happy. Seeing that brings me joy. Additionally, it's also rewarding to watch him improve and get better at things.*” (Participant D)

### Fostering family connections and parent-to-parent interaction

This theme includes the following sub-themes: Adjusting family dynamics, Opportunities for parent interaction, and Opportunities for exchanging information among parents.

The parents who participated in the event used it as a place to exchange information with other parents and to obtain information about support related to developmental disorders. Furthermore, the mothers of the participating parents talked about how they used horse riding to intentionally create opportunities to deepen the relationship between their child and a specific family member, or to create a distance between siblings and give them time to be apart.

“*My husband is not very involved in childcare, so the only time he’s really involved is on Sundays, when, despite his busy schedule, he has no choice but to come along. (.) It's rare for my child to interact with his father outside of playing games, and this is one of the few places where that happens. (.) So, I’m really glad we came here for this opportunity.*” (Participant B)

“*Other after-school programs usually have kids from the same city, which is quite common. (.) But when I think about my son going to high school, I realize we can't just focus on such a narrow circle. He’s been able to make friends with kids from other cities. (.) It’s been helpful because we can get valuable information from them.*” (Participant F)

## Discussion

### Experiences of connectedness with self, others, and society through EAATs

The four themes of long-term EAATs effects on children as perceived by their parents identified in this study may be interpreted as developmental stages. While presented as themes in the Results in accordance with the phenomenological approach, these can also be interpreted as sequential stages when viewed through the lens of Maslow (1954). Children first establish trust with the horse and staff within a psychologically safe relationship. This process can be understood as an experience in which body and mind become harmonized through physical coordination, corresponding to Maslow’s levels of physiological needs and the need for safety and security. Then, the children progress to self-exploration in a small community, gain recognition from others, and ultimately confront personal challenges independently. From this perspective, the four themes represent a trajectory of psychosocial growth, which parents recognize over the course of their child’s long-term participation in therapeutic horseback riding. Importantly, this developmental trajectory is not only abstract but is also grounded in concrete changes observed by parents, including improvements in communication, increased confidence, and, in some cases, participation in riding competitions. Nevertheless, the psychosocial aspects identified in this study highlight the importance of employing longitudinal or process-oriented approaches in future research to more comprehensively capture developmental trajectories over time.

**Figure 1.**
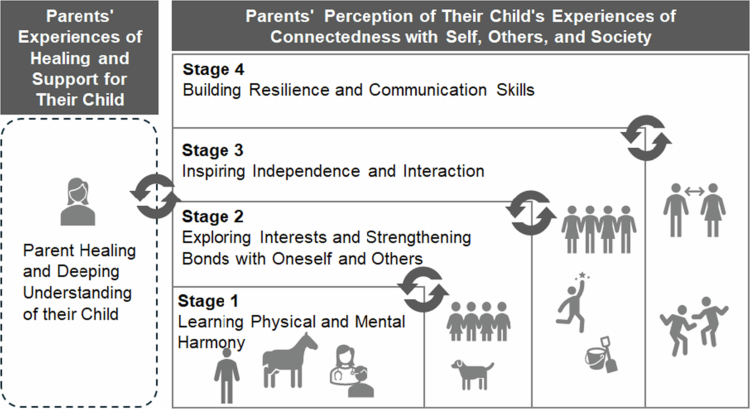
Parents’ experiences and their perception of their children’s experiences through long-term EAATs. (Icons created using Microsoft 365).

By progressing through these stages, children in EAATs were perceived to develop an embodied awareness of their own mind and body, which through growing interest in others, led them to consider how they wish to connect with society. This suggests the emergence of a dimension of social connectedness. Parents perceived these experiences as significant milestones that marked their child’s ongoing development (Figure 1).

In the first stage, children focus on learning about horseback riding in a psychologically safe environment provided by the staff. During this process, the staff infer and convey the horse's feelings to help children better understand it. A triangular relationship is nurtured, where the child connects with the staff and, through them, with the horse. Parents, observing from the outside, come to recognize new possibilities in their child’s development through their engagement with the horse. Parents also perceive gradual changes in how their child relates to the horse. These experiences were interpreted as “Parental Joy and Discovery in Their Child’s Growth.” Kojović et al. (2019) reported that in children with ASD, higher intensities of sensory issues are associated with more prominent social difficulties and lower adaptive functioning. In line with previous studies, bodily sensory difficulties have been identified as contributing to challenges in daily life. However, parents perceived that their children experienced a sense of security through connecting with both the staff and the horse. Moreover, by learning from the staff how to handle and engage with the horse, children acquired a foundation for the relationships that emerge during horseback riding.

In the second stage, the children become aware of how they wish to interact with others and begin putting that awareness into action within the small, safe community established in the first stage. This phase is regarded as a period in which children begin to present themselves to others. They also show interest in caring for dogs or younger children, displaying kindness, compassion, and sharing behaviors, as observed by their parents. The second and the third stages align with the social needs and the need for esteem in Maslow’s hierarchy.

In the third stage, the children become aware of how they are perceived by others as they begin to gain recognition. They present themselves in broader contexts. Although they may encounter small challenges, this stage is characterized by the children starting to address these challenges independently such as seeking advice from staff, rather than relying solely on their parents. Sumiya and Senju (2023) reported that many adolescents with ASD feel lonely because of the difficulties they experience when spending time, playing and seeking social interactions with their friends. In EAATs, the presence of other small animals, such as dogs; the existence of several small groups; the availability of trusted staff; and the presence of parents when needed may provide an environment that more readily accommodates each child’s own pace.

In the fourth stage, children are in a phase where they can take on tasks they previously avoided, including unpleasant ones, acting at their own pace and making decisions independently. The steps established up to the third stage have become routine, providing a foundation that expands the scope of the child’s daily activities. As they become capable of accomplishing more in everyday life, their confidence continues to grow. This stage may be regarded as corresponding to Maslow’s levels of esteem and self-actualization. Resilience is “the role of mental processes and behavior in promoting personal assets and protecting an individual from the potential negative effect of stressors” (Fletcher & Sarkar, 2013). In the present study, improvements were observed in children’s integrative coping abilities such as reorganizing their own plans in response to requests from younger peers or taking on challenges by expanding into experiences they had not previously engaged in. Events that had once been perceived by the children as sources of stress appeared to become manageable tasks. Through working on these tasks, parents perceived that their children were turning such experiences into personal resources for growth.

These findings suggest that EAATs may function as mediators of children’s broader psychosocial development. Such staged progress resonates with theories of social development, in which self-awareness and interpersonal engagement gradually expand into wider social participation. Moreover, these results do not depend solely on the act of riding itself, but also on the environment in which the horse is present, the tasks carried out there, and the communication that takes place in that setting. Such staged progress aligns with Vygotsky’s sociocultural theory, particularly the concept of the Zone of Proximal Development (ZPD) (Vygotsky, 1978), which posits that learning occurs in the zone between what a child can accomplish independently and what can be achieved with the guidance and support of more experienced others. This theory emphasizes that development takes place through social interactions within supportive environments, where scaffolding provided by instructors or peers enables children to internalize new skills and knowledge. In EAATs, interactions with horses, staff, and peers provide a scaffolded setting where children can gradually expand their self-awareness and interpersonal engagement, leading to broader social participation.

From this perspective, the sequential process from self-recognition to recognition of others and ultimately to social participation—observed in this study can be interpreted as an example of development within the ZPD, facilitated by the unique environment of therapeutic horseback riding.

### Parental growth and well-being in supporting their child’s participation in EAATs

Previous studies have reported that parents of children with ASD experience greater fatigue and lower QOL compared with parents of typically developing children. Moreover, such fatigue has been reported to influence parents’ feelings of loneliness (Kara et al., 2024). In the case of parents whose children attend EAATs, their involvement includes not only observing and supporting their child but also experiencing nature and refreshing themselves. In addition, opportunities to form relationships with other parents and to reflect on their distance and connection with their child and family may indirectly enhance the parents’ own well-being. Through participation in EAATs, children were able to enrich their process of engaging with society, while parents, by supporting this process, deepened their own sense of reassurance and understanding of their child’s growth. This suggests a mutually enriching relationship in which both the child and the parent develop together.

In this study, eight participants were mothers. This may have influenced the findings, as mothers also considered how EAATs could provide time to be with the father as a family member and opportunities for their own recovery from fatigue. Family roles and everyday relationships with their children may have affected our results.

### Practical implications for nursing and support services

It became evident that the development of children’s self-confidence evolved through multiple processes resembling a trajectory. Moreover, parents supporting their children also required support from staff members. The roles of various professionals in EAATs are important. Riding instructors create a psychologically safe environment and foster a triangular relationship between the child, staff, and horse. Physiotherapists and occupational therapists contribute by supporting motor skills and physical balance, thereby reinforcing children’s confidence to engage in social activities (American Hippotherapy Association, 2017; Cerquozzi et al., 2007). This study highlights that the psychosocial progress perceived by parents across different stages is partly attributable to the diverse professionals.

Based on the present findings, children engaging in long-term EAATs appear to progress through several developmental stages. Their trajectories do not necessarily follow a linear path but rather fluctuate, involving periods of regression or stagnation before further advancement. During these transitional phases, children may experience emotional instability, while parents are likely to face heightened anxiety and feelings of stagnation, potentially reinforcing the child’s instability in a reciprocal cycle. Such a developmental and transitional perspective on EAATs support has not been highlighted in previous research.

Gabrielsson et al. (2020) emphasized the development of psychiatric and mental health nursing in Sweden as a caring, reflective, recovery-oriented, health-promoting, and therapeutic practice that makes a difference, and they called for the further advancement of its transformative role in nursing. In this regard, psychiatric nurses could play a distinctive role by observing both psychological and physical changes, monitoring how these influence daily functioning and interpersonal relationships, and supporting parents in interpreting their child’s developmental progress. In particular, by anticipating the periods of instability that accompany developmental transitions, nurses could provide EAATs support from a transitional care perspective, thereby facilitating smoother adaptation for both children and their parents.

## Limitations

This study included 10 participants. The children of these participants presented with diverse conditions, including various comorbidities and disabilities. Given the long-term nature of their participation in EAATs, it is likely that their symptoms fluctuated over time. Although themes were extracted using a phenomenological approach, the parents’ perceptions were likely influenced by the specific periods during which they observed their children.

## Conclusion

From parents' perceptions of the psychosocial outcomes of therapeutic EAATs for children with ASD, four major themes were identified in the children’s experiences. In addition, three themes emerged from the parents’ own experiences of supporting their child’s participation.

Previous studies on EAATs have primarily focused on behavioral and psychological outcomes, which appear to correspond to the observable aspects identified in this study, particularly those manifested in behaviors from the second to the fourth stages. In contrast, the present findings reveal that parents perceived a longer-term, sequential developmental process in their children who participated in therapeutic horseback riding. Building on these findings, future research should broaden the participant pool and employ diverse methodologies to further examine this developmental trajectory. To support children’s psychosocial development and promote the health and well-being of their parents, this study also suggests nurses could play a distinctive role by observing both psychological and physical changes, monitoring how these influence daily functioning and interpersonal relationships, and supporting parents in interpreting their child’s developmental progress.

## Supplementary Material

Supplementary Material Supplementally Table. 1 (S1) Two-dimensional aspects of the long-term impact of EAATs for children with ASD as perceived by their parents, and sub-themes illustrated with participants' statement examples.

## Data Availability

The data that support the findings of this study are available from the corresponding author upon reasonable request.
