# Supplementary material for: Parental perceptions of the psychosocial outcomes of equine-assisted activities and therapies for children with autism spectrum disorder in Japan: a phenomenological study
Source: Int J Qual Stud Health Well-being. 2025 Nov 19;20(1):2585638. doi: 10.1080/17482631.2025.2585638 (PMC12636537; doi:10.1080/17482631.2025.2585638)
Supplement: Supplementary Material — Supplementally Table. 1 (S1) Two-dimensional aspects of the long-term impact of EAATs for children with ASD as perceived by their parents, and sub-themes illustrated with participants' statement examples. [file ZQHW_A_2585638_SM4553.docx]

**Supplementally Table. 1 （S1）Two-dimensional aspects of the long-term impact of EAATs for children with ASD as perceived by their parents, and sub-themes** **illustrated with participants' statement examples**

| **Dimension: Children's horseback riding experiences as perceived by parents** | | |
| --- | --- | --- |
| **Themes** | **Sub-themes (number of statements)** | **Examples of Statements** |
| **Learning Physical and Mental Harmony** | Enjoyment and sense of security in horseback riding (22) | “He doesn’t want to go to school, so he really dislikes when it’s time to leave. It seems like he just doesn’t want that moment to come, so unless we tell him, 'It’s time to go!' he won’t get ready. But with the pony sessions, it’s the opposite—he’ll be the one saying, ‘Dad, we’re going to be late! Let’s go!’” (Participant D) |
|  | Reassurance from professional staff support (16) | “When he feels tired or things get too hard for him, he chooses not to join. When something doesn’t go well, he can’t really explain what happened or how he feels — it just comes out as, “I don’t want to do it,” or “I’m not doing it.” But through his past experiences with the riding staff — who really took the time to match his pace and give him the right balance of support and space — I think he’s started to feel that there are people who understand him. And that’s given him a sense of security, like, “It’s okay — there are people who get me.” (Participant J) |
|  | Core stability (10) | “Ever since he started horseback riding, I’ve noticed changes in him around the end of first grade. He’s even been trying to apply what he does there at home. After a year of doing it, I feel like his core’s gotten a lot stronger. If he’s not paying attention, his body just goes all floppy, so when I say, ‘Back straight!’ he quickly straightens up. He’s still a bit weak, but I can see little by little he’s improving.” (Participant J) |
|  | Established as part of a daily routine (7) | “I think having these pony sessions twice a month probably helps my son switch his mindset. Rather than just staying at home all the time, coming here for horseback riding means he gets himself ready—mentally and with his things—and he does it on his own, more independently. Even though it’s only twice a month, the fact that he can do it as a routine, you know, I think that becomes one step toward living more independently in daily life.” (Participant I) |
|  | Improvement in smoothness of movement (8) | “I’ve been noticing steady changes in how he uses his body, and I really think he’s getting better. He used to always walk on his tiptoes, especially when he was nervous or anxious—his toes would just lift up. So, I always had this image that his ankles were kind of a weak point, and his body seemed tense all the time.  But since he’s been doing horseback riding, that’s really improved a lot. He can even keep his heels down now.　When he was little, people used to say he didn’t have a clear body image, but I think through riding, he’s developed one.　Before, all his movements were kind of awkward—like everything looked a bit stiff—but now he can throw a ball, adjust his strength, and judge distance better. His movements have become so much smoother.”　(Participant C) |
|  | Positive effects on work tasks (increased focus) (2) | “When we go to unfamiliar or fun places, my son tends to get really excited—like, waaah! Especially if friends are around, his energy really goes up, and, well, he’s often been told to calm down since he was little. But here, it’s more about interacting with the instructors rather than with friends, so he doesn’t get overly excited like that.” (Participant H) |
|  | Enhanced digestive function (1) | “And, you know, on the days after horseback riding, my son’s bowel movements get better. (…) He’s had so much constipation, ever since he was little. (…) On the days after riding, sometimes he’ll poop, like, three times. It’s really been a worry since he was small, but, yeah, I feel like the more he moves his body, the better he goes to the bathroom.” (Participant I) |
| **Exploring Interests and Strengthening Bonds with Oneself** | Expanded interest and empathy toward others (19) | “My son had only experienced being helped by others, not helping others himself. But, after coming here, caring for the animals became his main focus. I began to see new feelings in him, like wanting to take care of others and be kind to animals. It was the first time I had seen this side of him, and I believe it emerged through his direct interaction with the animals in horse therapy.” (Participant E) |
|  | Improved confidence through riding (16) | “You know, I feel like through horseback riding, he can see a side of himself that’s capable — a version of himself that can do things. At school, there seem to be a lot of things he struggles with or feels anxious about, but when he’s riding, I think he gets to meet the part of himself that can — the “capable him,” if that makes sense.”　(participant I) |
|  | Increased confidence through interaction with children of different ages (4) | “Up until now, my son was always the youngest one, but little by little, in horseback riding, there started to be kids younger than him. And I think, for him, being relied on like that was really good. (…) Being relied on, you know, like as the older brother. (…) And I think that made my son really happy. “(Participant A) |
|  | Broadened interest in horses and other animals (3) | “With animals, unless you take the initiative, they won’t really respond to you, right? I don’t think you get much back from them, but it still makes you want to reach out and interact with them yourself, and I think that’s a really good thing for my son.” (Participant I) |
|  | Improved planning skills (2) | “My son said he wanted to take the lead and do things himself, and, well, first of all, he really wanted to ride the horse. I guess it was that he thought the horse looked cool. But then, after he started riding, when he came to horseback riding, he realized, oh, besides riding, there’s also taking care of the horse. And he figured out that, you know, if he doesn’t take care of the horse, the riding practice won’t start. From there, he began to take the initiative — doing the cleaning, giving water, all that — and, yeah, I feel like he’s been spending the time with a real sense of responsibility.” (Participant B) |
|  | Opportunities to learn new tasks (1) | “My son has been able to do more things since coming here. (…) At home, of course, we don’t have him use a broom to clean, and there’s no chance to use a rake either, but here he’s been taught how to use a broom, how to use a rake, and even how to pick things up with a dustpan as part of the cleaning. And that, you know, has definitely carried over to the cleaning he does at school. And, well, horseback riding isn’t just about the fun of riding — it’s also about taking care of the horse, realizing that it’s a living being, and looking after it. So, I feel like my son has developed this sense that, yeah, you have to take care of living things, you have to value them.” (Participant H) |
| **Inspiring Independence and Interaction** | Expanded actions in daily life (9) | “In my child’s case, I think at first, he probably didn’t like cleaning up the horse’s poop. But now, he does it without complaining. I feel like that might have reduced his resistance to cleaning in general—even at home. So having stable cleaning as part of the schedule seems like a really good thing.” (Participant D) |
|  | Increased motivation for riding (8) | “My son says he wants to get a job working with horses, so, you know, he’s like, “If it’s in ○○ prefecture, maybe the horse racing training center?” That’s what he says. And I tell him, “It’s not that easy.” You know, if not that, then it would have to be somewhere else in the country, but, well, there aren’t horses everywhere. Only in certain places. So, I wonder, where would my son end up going? (…) It’s not like I explain future jobs to him through horseback riding, but just by being here he sees things — like when they change the horseshoes and the farrier comes, he watches that. And, you know, when a new horse arrives, there are also people who transport them, and he sees that too.” (Participant F) |
|  | Improved conversational and self-expression skills (7) | “It’s not just that he’s passive anymore—he’s started to take initiative and ask things like, “What should I do here?” or “Is it okay if I do that?” Not only at school, but also with people who aren’t his teachers or parents, he seems to be able to speak up on his own.” (Participant I) |
| **Building Resilience and Communication Skills** | Improved ability to cope with changing situations (16) | “My child never really had intense meltdowns to begin with. When he did, it was only in response to something completely unexpected, like, for example, if he accidentally walked into a spiderweb, then it would be a huge reaction. But now, he’s become much more flexible. Instead of a full-blown panic, it might just end with him being on the verge of tears or him being able to hold it together. For instance, if he was told he would be the first to ride but another child asked, 'Sorry, would you mind switching places with me?' my son would now say, 'Sure,' and let them go ahead.” (Participant C) |
|  | Expanded communication with diverse people through horseback riding (5) | “She mentioned how it was fun when older boys were kind to her and things like that. So, I feel like, for her, it wasn’t just the horses—the chance to connect with different people was also really good for her.” (Participant G) |
|  |  |  |
| **Dimension: Parental Experiences in Supporting Their Child’s Horseback Riding** | | |
| **Themes** | **Sub-themes (number of statements)** | **Examples of Statements** |
| **Parental Well-Being and Engagement Through Horseback Riding** | Parental refreshment (nature and healthy distance from the child) (9) | “I like animals, so being here helps me relax, even when I’m tired from work or housework. My son enjoys it too, so it’s a great stress reliever for both of us.” (Participant J) |
|  | Peace of mind from observing their child’s well-being (5) | “My son has swimming lessons in the morning, and after swimming he eats lunch and then comes straight here for horseback riding. So, yeah, in terms of daily rhythm it’s gotten busier, that’s true. (…) But when he’s at home, all I see is him playing video games, so horseback riding is probably the only place where I can actually see him really absorbed in something he likes. And, well, when it comes to watching my child grow like that, I feel like I’m also spending really fulfilling time.”(Participant D) |
|  | Reassurance from staff's professional support (specifically for parents) (6) | “By coming here, I can get advice from the teachers. And from my point of view, there are parts of my son that feel really tough or difficult to cope with. (…) Having a place where I can talk if something comes up, a place where I can say what I need to say —that’s something that’s been really important for us, ever since the early therapy days when I’d always talk with other moms.” (Participant C) |
|  | Increased interest in horseback riding through the child’s involvement (2) | “My son wanted to ride horses, so he even tried riding at places like tourist ranches. And just watching him made me feel like maybe I wanted to try riding too. (…) For me, I’ve always been kind of bad with animals — we never had pets at home, and I was even scared of dogs and cats. But through this, I think I’ve started to take an interest in animals, maybe even to like them.” (Participant F) |
|  | Stability in parents' routines through transport arrangements (1) | “Since I was a full-time housewife, once my son went off to horseback riding, I would just kind of flop down and relax, you know? But when I thought about moving in rhythm with him, to match his schedule, (…) I feel like I’ve actually become more active myself.” (Participant F) |
| **Parental Joy and Discovery in Their Child’s Growth** | Joy in witnessing the child’s growth as a parent (8) | “There are competitions here as well. The staff at this facility make sure to praise and encourage the children, giving out awards to everyone. When this happens, he looks genuinely happy. Seeing that brings me joy. Additionally, it's also rewarding to watch him improve and get better at things.” (Participant D) |
|  | Discovering new strengths in their child as a parent (impressed) (8) | “He’s still not very good at moving his body. And when I see little kids around our neighborhood, like 3- or 4-year-olds, starting to ride bikes or jump rope, I can’t help but think, “Why can’t he do that?” Even though I don’t say it out loud, I still think it. But then, I start to notice the things he’s working hard on. And because of that, I think, ‘Oh, he’s definitely growing,’ and it reminds me that there’s no way he won’t grow. It makes me think I should take things a little slower, and approach things in a way that suits him better. Every time we come here, I end up thinking this way.” (Participant H) |
| **Fostering Family Connections and Parent Interaction** | Adjusting family dynamics (6) | “My husband is not very involved in childcare, so the only time he’s really involved is on Sundays, when, despite his busy schedule, he has no choice but to come along. (...) It's rare for my child to interact with his father outside of playing games, and this is one of the few places where that happens. (...) So, I’m really glad we came here for this opportunity.” (Participant B) |
|  | Opportunities for parent interaction (4) | “But I feel like between teachers and parents, our feelings have, you know, gotten a bit closer, like we’ve come to understand each other better. I think it’s helped us see more clearly what exactly our children struggle with, and in what situations. (…) And there’s also this sense of connection, like, among us parents.” (Participant J) |
|  | Opportunities for exchanging information among parents (3) | “Other after-school programs usually have kids from the same city, which is quite common. (...) But when I think about my son going to high school, I realize we can't just focus on such a narrow circle. He’s been able to make friends with kids from other cities. (...) It’s been helpful because we can get valuable information from them.” (Participant F) |
